# Syncytial apoptosis signaling network induced by the HIV-1 envelope glycoprotein complex: an overview

**DOI:** 10.1038/cddis.2015.204

**Published:** 2015-08-06

**Authors:** R Nardacci, J-L Perfettini, L Grieco, D Thieffry, G Kroemer, M Piacentini

**Affiliations:** 1 Laboratory of Cell Biology and Electron Microscopy, National Institute for Infectious Diseases, IRCCS ‘L. Spallanzani', Rome, Italy; 2 Cell Death and Aging Team, Gustave Roussy, Villejuif, France; 3 Laboratory of Molecular Radiotherapy, INSERM U1030, Gustave Roussy, Villejuif, France; 4 Gustave Roussy, Villejuif, France; 5 Université Paris Sud—Paris 11, Villejuif, France; 6 Aix-Marseille Université, Marseille, France; 7 TAGC—Inserm U1090, Marseille, France; 8 Institut de Biologie de l'Ecole Normale Supérieure (IBENS), Paris, France; 9 UMR 8197 Centre National de la Recherche Scientifique (CNRS), Paris, France; 10 INSERM U1024, Paris, France; 11 Institut Curie, Paris, France; 12 Clinical Operational Research Unit, University College London, London, UK; 13 INRIA Paris-Rocquencourt, Rocquencourt, France; 14 Equipe 11 labellisée par la Ligue Nationale contre le Cancer, Centre de Recherche des Cordeliers, Paris, France; 15 Université Paris Descartes/Paris V; Sorbonne Paris Cité, Paris, France; 16 INSERM,U1138, Villejuif, France; 17 Metabolomics and Cell Biology Platforms, Gustave Roussy, Villejuif, France; 18 Pôle de Biologie, Hôpital Européen Georges Pompidou, AP-HP, Paris, France; 19 Department of Biology, University of Rome ‘Tor Vergata', Rome, Italy

## Abstract

Infection by human immunodeficiency virus-1 (HIV-1) is associated with a progressive decrease in CD4 T-cell numbers and the consequent collapse of host immune defenses. The major pathogenic mechanism of AIDS is the massive apoptotic destruction of the immunocompetent cells, including uninfected cells. The latter process, also known as by-stander killing, operates by various mechanisms one of which involves the formation of syncytia which undergo cell death by following a complex pathway. We present here a detailed and curated map of the syncytial apoptosis signaling network, aimed at simplifying the whole mechanism that we have characterized at the molecular level in the last 15 years. The map was created using Systems Biology Graphical Notation language with the help of CellDesigner software and encompasses 36 components (proteins/genes) and 54 interactions. The simplification of this complex network paves the way for the development of novel therapeutic strategies to eradicate HIV-1 infection. Agents that induce the selective death of HIV-1-elicited syncytia might lead to the elimination of viral reservoirs and hence constitute an important complement to current antiretroviral therapies.

## Facts:

- HIV-1 infection involves the apoptotic destruction of infected cells (‘direct killing') and of non-infected cells many of which are immunologically relevant (‘bystander killing').
- The by-stander killing operates by different mechanisms, one of which involves the formation of syncytia.
- HIV-1-Env-induced syncytium formation leads to apoptosis via a highly complex signaling network.
- In spite of the significant improvements achieved with the introduction of combination antiretroviral drug therapy for the management of HIV-1 infection, we are still far from being able to prevent the infection or to eradicate the virus from its reservoirs.

## Open Questions:

- Could the modulation of syncytia cell death pathway represent a potential therapeutic strategy for counteracting HIV-1 infection?
- On theoretical grounds, might agents that induce the selective death of HIV-1-elicited syncytia might lead to the elimination of viral reservoirs and hence constitute an important complement to current antiretroviral therapies?
- Could the modulation of apoptosis induced by HIV-1 (rather than the infection by HIV-1 itself) provide some clinical benefit for the control of HIV-I-induced pathogenesis?

Infection with human immunodeficiency virus type 1 (HIV-1) causes a progressive loss of CD4^+^ T cells that leads to the development of acquired immunodeficiency syndrome (AIDS). Apoptosis occurs via two distinct pathways, an intrinsic and an extrinsic pathway. The extrinsic (or external) pathway is initiated by the binding of ligands such as Fas ligand (FasL), TNF and TRAIL/Apo-2 ligands to their death receptors FAS/CD95, TNFR1, DR4 and DR5. The pathway relies on the activation of caspases 8 and 10, which in turn activate the effector caspases 3 and 7. The intrinsic (or internal) pathway is initiated by the disruption of the mitochondrial membrane, resulting in the release of cytochrome c, regulated by the bcl-2 family of proteins.^1^ The apoptotic pathways induced by HIV proteins are components of the intrinsic as well as the extrinsic apoptotic pathways.

T lymphocyte depletion associated with HIV-1 infection involves the death of infected cells, but is mainly due to the selective destruction of uninfected cells.^2, 3, 4, 5^ This process, also known as by-stander killing, can be induced by viral incomplete reverse transcripts or proteins, such as the HIV-1 envelope complex (Env), Tat, Nef, Vpu and Vpr,^6, 7^ by excessive T lymphocyte activation^8^ or by abortive infection^6, 9^ of immune cells. Loss of T lymphocytes from HIV-1 infected individuals may involve distinct modalities of cellular demise including necrotic, apoptotic, autophagic or pyroptotic death.^6, 7, 9, 10^

Apoptosis induction by viral proteins provides a plausible explanation for most of the phenomena observed during HIV-1 infection that allow the progression to AIDS.^11, 12^ The role of the Env glycoprotein in this process is particularly important.^4, 13^ The Env glycoprotein (gp) precursor protein (gp160) undergoes proteolytic maturation so as to generate gp41 and gp120 proteins. Binding of soluble gp120 to its receptor CD4 and its co-receptors, namely either of the two chemokine receptors CXCR4 or CCR5, which are expressed at the membrane of HIV-1 target cells, can induce apoptosis of uninfected cells.^14^ The membrane-anchored Env gp120/gp41 complex present at the surface of HIV-1-infected cells can interact with co-receptors and induce apoptosis of uninfected cells by at least three mechanisms.^4^ First, the interaction between Env- and CD4/CXCR4- (or CD4/CCR5-) expressing cells can trigger the so-called hemifusion procession resulting in the exchange of lipids between the plasma membranes of the transiently interacting cells, culminating in the killing of uninfected cells. This process has been reported as a ‘kiss of death'. Second, virus-infected cells that are on the point of undergoing apoptosis may fuse with CD4-expressing cells, in which case apoptosis is rapidly transmitted from one cell to the other and precipitates the death of uninfected target cells. This process, which occurs in a non-cell autonomous manner, has been referred to as ‘contagious apoptosis'.^15^ Third, the interaction between the two cells may induce full-blown cellular fusion (also known as cytogamy) and hence results in syncytium formation. Subsequently, nuclear fusion (karyogamy) occurs within the syncytium, which succumbs to apoptosis. This type of death, which is known as ‘syncytial apoptosis', is frequently detected during HIV-1 infection *in vitro* and *in vivo*, in tissues from HIV-1-infected patients.^16^

Env variants interacting with CD4/CXCR4 (rather than those having a preference for CD4/CCR5) are mostly encoded by syncytium-inducing HIV-1 strains, and a strong correlation between CD4+ T-cell decline and infection by syncytium-inducing HIV-1 variants has been established.^16^ Many distinct viruses express on their plasma membrane surface glycoproteins that interact with receptors on target cells and facilitate virus–cell fusion. Upon viral replication, infected cells often expose virus-encoded fusion proteins on their surface, thereby enabling infected cells to fuse with uninfected cells and to generate multinucleated syncytia. The HIV-1 glycoprotein Env enables the virus to attach and fuse with target cells to infect them. When Env is still incorporated in the plasma membrane of the infected cell, it drives the adhesion between virus producer cell and target cells, which gives rise to the formation of the so-called virological synapse.^17^ This process can result in cell-to-cell fusion thus generating syncytia. Cell–cell fusion is a highly heterogeneous process and is the result of specific features in donor and target cell types; it results in diverse outcomes with potentially significant consequences for the success or failure of the HIV-1 infection. The ability of viruses to induce syncytia is generally assumed to be indicative of the viruses being transmitted through a cell-to cell pathway, without exposure to the extracellular milieu, thus allowing the virus to spread rapidly and to evade the immune response of the host. Cell-to-cell transmission overcomes barriers introduced in the donor cell at the level of gene expression and surface retention by the restriction factor tetherin.^18, 19, 20^ However, to date, it remains unclear whether syncytia are beneficial or detrimental for virus replication.

These ‘giant multinuclear cells' were detected in several tissues, in particular the brain and lymphoid organs of HIV-1-infected individuals, even when these patients were in the asymptomatic phase of the disease. Characterization of the phenotypic markers of such multinucleated cells suggested that syncytia may originate from the fusion of monocytes, lymphocytes and/or dendritic cells.^20^ The process of syncytia formation constitutes a hallmark of HIV-1 infections in humans, monkey models and mouse models.^21, 22^ Moreover, syncytia formation has been linked to HIV-1 pathogenesis and progression to AIDS, in as far as viruses with a syncytia-inducing (SI) phenotype surge in a late phase of the phase associated with rapid CD4^+^ T-cell decline.^23^ Because death of syncytia by apoptosis is commonly observed in co-cultures of infected and uninfected cells, cell–cell fusion is a potential mechanism underlying CD4^+^ T-cell depletion.^4, 5, 24, 25^ A positive correlation between CD4^+^ T-cell decline and infection by syncytium-inducing HIV-1 or SIV-1 variants has been established *in vitro*^26, 27, 28^ and, more importantly, *in vivo*, in humans with AIDS.^29, 30^ A co-culture system utilizing HIV-1 Env-expressing cells and uninfected CD4^+^ T cells provided the first evidence for the role of Env-mediated apoptosis in T-cell depletion more than two decades ago.^31^ Since then, the characterization of the mechanisms of HIV-1-induced apoptosis has advanced considerably, leading to the implication of unexpected molecular players including purinergic receptors, the DNA damage response, activation of the TP53 tumor suppressor protein, as well as mitochondrial alterations, in HIV-1-triggered cell loss. The present review will focus on the proapoptotic signaling network involved in the induction and execution of syncytial apoptosis.

## Purinergic Receptor Activation Favors Syncytial Apoptosis Triggered by HIV-1 Env

**Figure 1:**
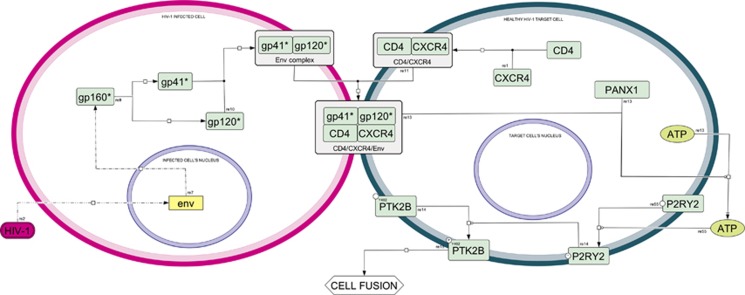
Syncytial apoptosis map—cell fusion. Interaction between a HIV-1-infected cell (purple compartment) and a CD4^+^ healthy cell (green compartment). This part includes all the reactions leading to the activation of P2RY2 and PTK2B proteins, determining cell-to-cell fusion. Nuclei are denoted with blue contours, proteins are colored in green, genes are colored in yellow, viruses are colored in purple. Asterisks indicate non-official HGNC names. Please see Supplementary Information for more detailed map notion

Purinergic receptors are membrane-anchored receptors that bind extracellular nucleotides such as adenosine triphosphate (ATP) and uridine triphosphate (UTP). Purinergic receptors are expressed on numerous HIV-1 target cells (including T cells, macrophages and dendritic cells) and are involved in multiple cellular functions including proliferation, differentiation, membrane pore formation, cytokine secretion, chemotaxis and cell death. They participate in various physiological and pathological processes including cancer, inflammatory-related disorders and infectious diseases.^32, 33, 34^ Purinergic receptors are classified into ionotropic P2RX and metabotropic P2RY receptors. P2RX receptors (numbered as P2RX1 to P2RX7) are ligand-gated ion channels that form trimeric structures utilizing individual subunits. P2RY receptors are G protein-coupled receptors with eight subtypes (P2RY1, P2RY2, P2RY4, P2RY6, P2RY11, P2RY12, P2RY13 and P2RY14). Several studies indicate that the binding of HIV-1 to host cell increases the intracellular free calcium concentration, suggesting a potential role for membrane receptors or channels at the early stage of viral infection.^35, 36, 37, 38, 39^ In this context, we initially demonstrated that purinergic receptors, pericellular nucleotide release (such as ATP) and related signaling pathways have a key role in HIV-1 infection by contributing to viral entry into host cells, as well as possibly to other steps of the viral life cycle. Thus, we discovered that approximately 1 h after the binding of the HIV-1 envelope to cellular receptors ATP is released from target cells (Figure 1). Then, extracellular ATP stimulates purinergic signals that favor HIV-1 infection by facilitating hemifusion and fusion processes mediated by HIV-1 Env. ATP release is at least in part mediated by mechanosensitive pannexin-1 hemichannel. Pannexin-1 (PANX1) was found enriched at the contact site between HIV-1-infected lymphoblasts and uninfected lymphoblasts^40^ and ATP that is rapidly released from HIV-1 target cells during the infectious process contributes to viral entry. We found that several purinergic receptors (such as P2RX4, P2RX7, P2RY1, P2RY2 and P2RY6) may contribute to the early steps of HIV-1 infection. We detected after 3 h and half of coculture that the purinergic receptor P2RY2 accumulates at virological synapse formed by interacting cells. Moreover, we demonstrated that P2RY2 coordinates the formation of a polyprotein complex that activates protein tyrosine kinase 2 beta (PTK2B, also known as PYK2), by inducing its (auto)phosphorylation on tyrosine 402 (PTK2BY402*) and controls plasma membrane depolarization (PMD) of target cells as a necessary step for subsequent HIV-1 Env-mediated hemifusion and fusion processes.^40^ The implications of pannexin-1 and purinergic receptors in the early steps of infection of human primary CD4^+^ T lymphocytes and macrophages have been confirmed by others.^41, 42^ Moreover, the involvement of purinergic receptor signaling in later stages of the viral life cycle has recently been proposed.^42, 43^ In addition purinergic receptors, which are expressed in HIV-1-infected perivascular macrophages, have been implicated in HIV-associated neurocognitive disorder through the dysregulation of glutamatergic signaling and the reduction of dendritic spine density on neurons.^44^ Thus, purinergic receptors could provide an attractive therapeutic target for HIV therapy. Targeting host-intrinsic (rather than virus-encoded) receptors would have the intrinsic advantage of avoiding any kind of resistance mechanisms linked to the remarkable capacity of the virus to mutate.^34^ Purinergic receptor antagonists (such as suramin, PPADS and oxidized ATP) potently prevent the entry of X4-tropic HIV, R5-tropic HIV or therapy-resistant HIV-1 mutants in T leukemia cells, as well as in activated primary T lymphoblasts and primary monocyte-derived macrophages.^40^ These results were recently confirmed,^45^ highlighting that the blockade of purinergic signaling pathways can confer immune protective effects and open the way for novel antiretroviral therapies.

## HIV-1 Env-induced Nuclear Fusion Triggers Pro-apoptotic DNA Damage

**Figure 2:**
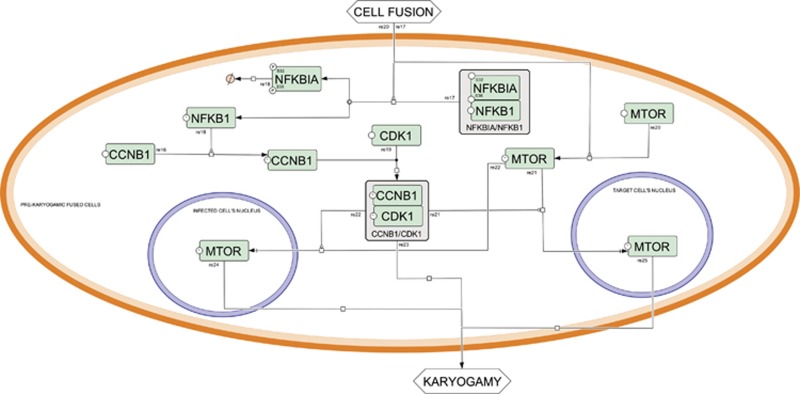
Syncytial apoptosis map—pre-karyogamic syncytium. Events occurring within a pre-karyogamic syncytium (orange compartment). In particular, activation of NFKB1 and mTOR pathways are reported as main determinants of nuclear fusion (karyogamy). Nuclei are denoted with blue contours, proteins are colored in green, genes are colored in yellow. Please see Supplementary Information for more detailed map notion

After 10 h of coculture, interacting cells fuse their cytoplasm (also known as cytogamy) and arising syncytia exhibit two intact nuclei that defined the prekaryogamic stage of syncytial apoptosis (Figure 2). During this stage, pro-apoptotic signaling pathways emanating from plasma membranes stimulate the transcription factor NFKB1 (nuclear factor of kappa light polypeptide gene enhancer in B-cells 1, NF-kappaB). This is mediated by the activation of the inhibitor of NFKB1, namely NFKBIA (nuclear factor of kappa light polypeptide gene enhancer in B-cells inhibitor, alpha, (IkappaBalpha, IκB), resulting in the inhibitory phosphorylation of serines 32 and 36 of NFKBIA (NFKBIAS32*/S36*), resulting in turn in NFKBIA degradation, dissociation of NFKB1 and its translocation into the nucleus.^25, 46, 47^ NFKB1 transactivation induces in a transient manner the upregulation of cyclin B, thereby triggering an abortive entry into the prophase of mitosis resulting in the disassembly of the nuclear envelope. This latter process facilitates karyogamy, namely fusion of nuclei within the heterokaryon.^16, 46^ After 18 h of coculture, karyogamic syncytia accumulate DNA lesions, as revealed by the detection of phosphorylated H2AFX (H2A histone family, member X, H2AX) on serine 139, H2AXS139* (also known as *γ*-H2AX) in so-called DNA damage foci.^48^ In lymphoid and brain tissue sections from HIV-1-infected patients, more than 60% of multinuclear giant cells (syncytia) stained positively for *γ*-H2AX foci, suggesting that syncytium formation is indeed linked to the activation of a disease-relevant DNA damage response.

**Figure 3:**
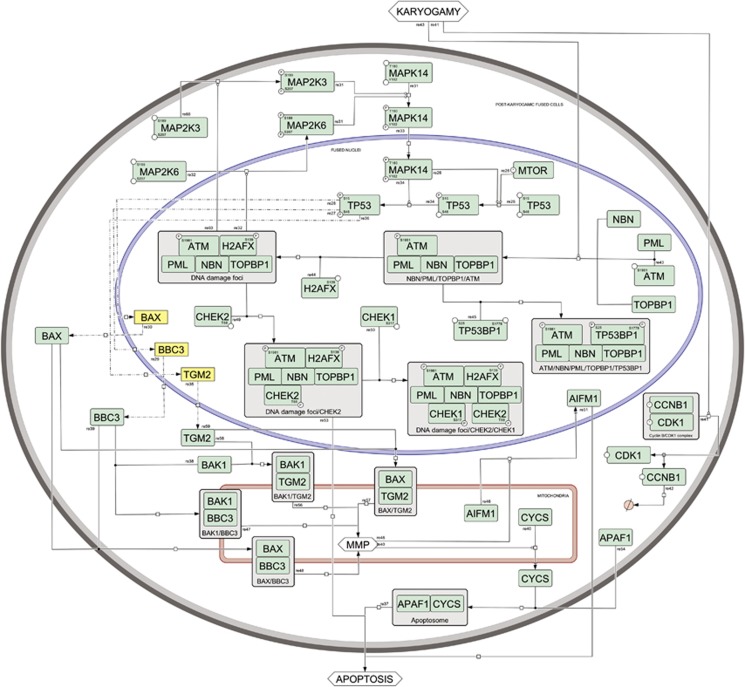
Syncytial apoptosis map—post-karyogamic syncytium. Reactions causing apoptosis in a post-karyogamic syncytium (gray compartment with dark background). Activated TP53 protein is the main actor of this step, leading to mitochondrial membrane permeabilization and apoptosome formation, and subsequently to syncytial apoptosis. Nuclei are denoted with blue contours and mitochondria with red contours, proteins are colored in green, genes are colored in yellow. Please see Supplementary Information for more detailed map notion

After karyogamic stage (Figure 3), the nuclei from Env-elicited syncytia accumulate promyelomonocytic leukemia (PML) nuclear bodies (PML-NBs).^49^ PML-NBs are defined by the presence of a protein called PML, a prominent onco-suppressor that is inactivated in some leukemias.^50^ PML proteins aggregate as a result of cellular and nuclear fusion and activate the ataxia telangiectasia mutated (ATM) kinase. ATM is a potent tumor suppressor protein, previously implicated in the control of TP53 (tumor protein p53) in tumors,^51, 52^ and has a major role in HIV-1 infection both *in vitro* and *in vivo*.^4, 47, 53^ Karyogamic syncytia exhibit the activating phosphorylation of ATM on serine 1981 (ATMS1981*) within their nuclei.^48^ PML and ATM colocalize and co-immunoprecipitate in a molecular complex, which contains other proteins involved in the DNA damage response including TOPBP1 (topoisomerase II*β*-binding protein 1) and NBN (Nijmegen breakage syndrome 1, NBS1). PML depletion reduced the abundance of TOPB1, NBN and ATM, whereas TOPBP1 depletion only reduced the expression of NBN and ATM (but not that of PML). Depletion of either TOPBP1 or NBN reduced the size of PML bodies, whereas ATM depletion had no such effect. Knockdown of PML, TOPBP1 and NBN all had a similar inhibitory effect on ATM phosphorylation, the phosphorylation of ATM substrates, and downstream events leading to apoptosis. These results suggest that PML, TOPBP1, NBN and ATM interact within a syncytium-associated nuclear complex in which TOPBP1 and NBN act as functional links between PML and ATM.^50^

In the course of HIV-1 infection, another protein involved in the DNA damage response is activated: the tumor suppressor protein, tumor suppressor p53-binding protein 1 (TP53BP1, 53BP1). TP53BP1 is a BRCA1 carboxy terminal (BRCT) repeat protein with an important role in the DNA damage response. This protein is recruited to DNA damage foci within syncytia and undergoes an ATM-dependent activating phosphorylation.^54^ Normally diffusely distributed through the nucleus during interphase, TP53BP1 is recruited to sites of DNA lesions upon a DNA damage response; here it interacts with DNA double-strand breaks and many proteins involved in DNA damage, repair and checkpoint signaling, including BRCA1, RAD51, MRE11/RAD50/NBN, ATM and *γ*-H2AX, to constitute DNA damage-inducible foci. In karyogamic nuclei from Env-elicited syncytia, TP53BP1 colocalizes partially with PML and ATM, the two components of the DNA damage response that mediate apoptosis induced by the HIV-1 envelope. ATM-dependent phosphorylation of TP53BP1 on serines 25 and 1778 (TP53BP1S25* and TP53BP1S1778*) occurs at these DNA damage foci. TP53BP1S25* was also detected in syncytia present in the lymph nodes or frontal brain sections from HIV-1-infected carriers, as well as in peripheral blood mononuclear cells from HIV-1-infected individuals, correlating with viral load.^54^ Knockdown of TP53BP1 caused HIV-1 envelope-induced syncytia to enter abnormal mitoses, leading to their selective destruction through mitochondrion-dependent and caspase-dependent pathways. We observed that aggregation of phosphorylated TP53BP1 depends on karyogamy but not on apoptosis. Thus, ATM is required for TP53BP1 phosphorylation but dispensable for its aggregation within the nuclei of karyogamic, indicating that TP53BP1 can interact with DNA in the absence of phosphorylated H2AX.

Syncytia also manifest activating phosphorylations of the checkpoint kinases 1 and 2 (CHEK1 and CHEK2), and both CHEK1 and CHEK2 colocalize with *γ*-H2AX foci.^55^ CHEK1 is activated by ATR while CHEK2 is activated by ATM, although some crosstalk between these pathways occurs. CHEK2 is activated by phosphorylation on threonine 68 (CHEK2T68*) during the karyogamic stage of syncytia and is found within the nucleus, colocalizing with *γ*-H2AX-positive foci. CHEK2 might operate upstream of CHEK1 and downstream of DNA damage response (PML, TOPB1, ATM). CHEK2 is a pro-apoptotic signal transducer in syncytia, but it can also activate an anti-apoptotic pathway involving the phosphorylation of CHEK1 on serine 317 (CHEK1S317*). The siRNA-mediated knockdown of CHEK2, but not the depletion of CHEK1, inhibits mitochondrial outer membrane permeabilization and subsequent syncytial apoptosis. Depletion of PML, TOPBP1, NBN or ATM inhibits the activating phosphorylation of CHEK2. Altogether, these results indicate that CHEK2 (but not CHEK1) participates in the DNA damage-elicited pro-apoptotic cascade that leads to the demise of Env-elicited syncytia.^55^

## Contribution of TP53 to HIV-1 Env Mediated Pro-apoptotic Signaling

TP53 is involved in numerous biological processes such as cell-cycle progression, DNA repair, cell metabolism and cell death, which are mainly related to its transcriptional activity.^56^ Pharmacological and genetic inhibition of TP53 demonstrated that the transcriptional activities of TP53 are involved in the induction of Env-elicited syncytial apoptosis.^46^ Post-translational modifications of TP53 (including phosphorylation, acetylation and sumoylation) are known to increase its transactivating function (by inhibiting its interaction with the ubiquitin ligase MDM2),^57^ to activate the transcription of several pro-apoptotic BCL-2-related genes (such as BAX and BBC3)^58^ and to induce apoptosis in various stress situations.^59^ After HIV-1 Env-elicited karyogamy, the transcription factor TP53 is phosphorylated on serine 15 (TP53S15*) and on serine 46 (TP53S46*) by mammalian target of rapamycin (mTOR) and mitogen-activated protein kinase 14 (MAPK14, also called p38-α) (Figure 3). Both phosphorylation reactions are detectable in HIV-1 carriers.^47, 53^ Accumulated in the nuclei of karyogamic syncytia, mTOR is a member of the phosphatidylinositol 3 kinase-related kinases (PIKK) family that has a role in the balance between protein synthesis and degradation. After its translocation from the cytoplasm to the nucleus, mTOR induces the phosphorylation of TP53 on serine 15 (TP53S15*).^60, 61^ Cytoplasmic MAPK14 undergoes an activating phosphorylation on threonine 180 and tyrosine 182 (MAPK14T180/Y182*) before karyogamy, and then translocates from cytoplasm into karyogamic nuclei and phosphorylates TP53 on serine 46, thus increasing its transactivating function.^53^ Inhibition of MAPK14 completely abolishes syncytial apoptosis, and activated, phosphorylated MAPK14 is detected in the same cells that contain TP53S15*- and TP53S46*-positive nuclei, *in vitro, ex vivo* and *in vivo*.^53, 61, 62^ Syncytia containing MAPK14T180/Y182* were observed in lymph-node biopsies from HIV-1 carriers, in the brain of patients with HIV-1-associated dementia, as well as in cocultures of HeLa expressing the HIV-1 envelope (Env) with HeLa cells expressing CD4. MAPK14 is the principal kinase acting on serine 46 of TP53 while both mTOR and MAPK14 phosphorylate TP53 on serine 15.^4, 48, 53^ TP53S46* is influenced indirectly by ATM kinase through the signaling pathway that involved MAPK14 and its upstream regulators, MAP kinase kinases 3 and 6 (MAP2K3/ MAPK6). Activating phosphorylations of MAPK3 or MAPK6 on serines 189 or 207 (MAPK3/6S189/207*) were also detected on HIV-1 Env-elicited syncytia.^53^ Accordingly, chemical or genetic inhibition of ATM prevented the activation of MAP2K3/MAP2K6 and MAPK14. In fact, ATM inhibition reduced TP53S46* (which is mediated by MAPK14), but did not affect TP53S15* (linked to karyogamy).^48^ Gene expression analysis revealed that 82 TP53 target genes are modulated after the induction of HIV-1 Env-mediated signaling pathways. Among these genes, pro-apoptotic Bcl-2 family numbers, namely *BBC3*(*PUMA*) and *BAX*, are transcribed.^46^ In addition, the expression of transglutaminase type 2 (TGM2, transglutaminase 2), another TP53 inducible gene known to act as a multifunctional BH3-only protein at the mitochondrial level^63^ is detected in syncytia.^24, 25^ Activation of these proteins is known to induce mitochondrial membrane permeabilization (MMP) and to cause apoptosis, suggesting that TP53-dependent signals elicited by HIV-1 Env may converge to mitochondria and induce the release of apoptogenic factors (such as cytochrome *c* (CYCS) and mitochondrion-associated apoptosis inducing factor 1 (AIFM1, AIF)).

## MMP and Apoptotic Demise

HIV-1 Env-induced syncytial apoptosis is characterized by caspase activation and MMP (Figure 3).^64^ Twenty-four hours after the interaction between HIV-1-infected cells and uninfected cells, the TP53-inducible BH3-only protein BBC3 is upregulated in syncytia,^58^ leading to the activation of the proteins BAX and BAK1 (by inducing a pro-apoptotic conformation change of these proteins and favoring their insertion into the outer mitochondrial membrane), thereby stimulating the dissipation of the mitochondrial inner membrane potential and the release of apoptogenic factors from the intermembrane space to the cytosol through the permeabilized outer mitochondrial membrane.^65^ Release of cytochrome *c* (CYCS) from the mitochondrial inter-membrane space triggers the oligomerization of the apoptotic peptidase activating factor 1 (APAF1) and the procaspase-9 into a caspase activation multi-protein complex (also known as ‘apoptosome') allowing the proteolytic maturation of procaspase-9 and procaspase-3 into active caspases (CASP9 and CASP3) and the induction of apoptosis.^47, 53, 58, 60, 66, 67^

Moreover, Env-elicited syncytial apoptosis is characterized by the mitochondrial release of caspase-independent death effectors such as apoptosis inducing factor (AIFM1),^68^ a flavoprotein NADH oxidase that translocates to the nucleus where it interacts with DNA and forms the cyclophilin-dependent ‘degradosome', a DNA degradation complex.^64^ This proapoptotic signaling pathway may be in some circumstances be repressed by antiapoptotic factors induced by other HIV-1 proteins with the result of contributing to immune cell survival, promoting viral replication and potentially leading to the establishment of viral reservoirs.^69^

## Concluding Remarks

A positive correlation between CD4^+^ T-cell decline and infection by syncytium-inducing HIV-1 or SIV-1 variants has been established *in vitro* and, more importantly, *in viv*o, in humans with AIDS. HIV-1-Env-induced syncytium formation leads to apoptosis via a highly complex signaling network that we have outlined by using a system biology approach in this interactive model map.

**Figure 4:**
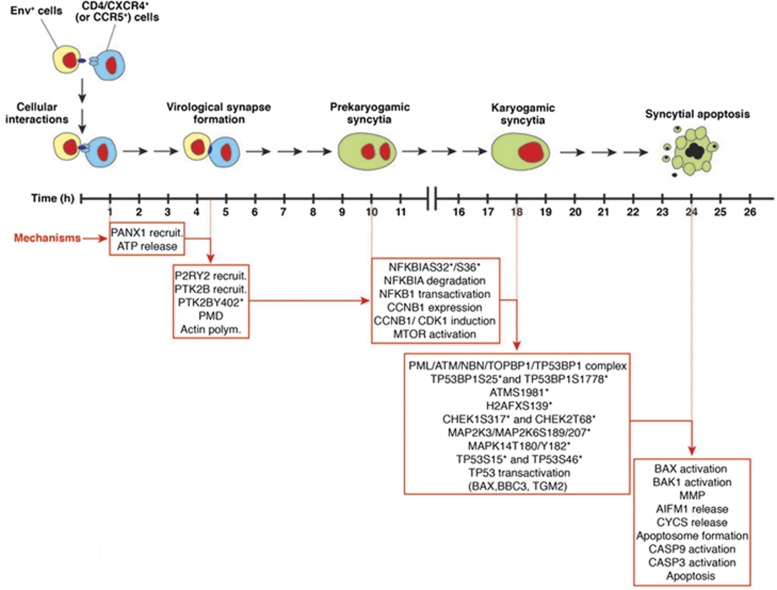
Timeline of syncytial apoptosis. Distinct stages that lead to syncytial apoptosis are shown. The timing and the cellular mechanisms involved in this atypical cell killing are also indicated

The data summarized in this study derive from initial observations carried out in *in vitro* models of syncytial apoptosis, in particular a co-culture system involving HeLa cells transfected with the HIV-1^LAI^ Env gene and HeLa cells transfected with CD4. These findings have been confirmed and validated in human tissues obtained from HIV-1-infected individuals. Our findings have clarified that apoptosis occurs only in syncytia, as a result of a timely regulated stepwise process (Figure 4). After an initial stage during which the cells contain well separated nuclei with intact envelopes, nuclear fusion (karyogamy) occurs. This nuclear fusion is the expression of an abortive entry into the mitotic prophase stimulated by the transient activation of the cyclin B dependent kinase-1 (CDK1), accompanied by the permeabilization of the nuclear envelope, presumably due to the CDK1-mediated phosphorylation of lamin (which favors lamin depolymerization and hence disassembly of the nuclear envelope). It is only after karyogamy has occurred that syncytia die from apoptosis.

The interactive model we propose here represents an important tool for future studies investigating the effects of new drugs or combination of already existing molecules on this pathway of cell death which have such a key role in the HIV-1-induced pathogenesis.

On theoretical grounds, agents that induce the selective death of HIV-1-elicited syncytia might lead to the elimination of viral reservoirs and hence constitute a complement to current antiretroviral therapies.

## Abbreviations

HIV-1: human immunodeficiency virus-1
AIDS: acquired immunodeficiency syndrome
Env: HIV-1 envelope complex
gp: Env glycoprotein
CXCR4: CD4 co-receptor in lymphotropic ENV variants
ATP: adenosine triphosphate
UTP: uridine triphosphate
CCNB1: cyclin B1
CDK1: cyclin-dependent kinase 1
PANX1: pannexin-1
P2RX: ionotropic purinergic receptors
P2RY: metabotropic purinergic receptors
PTK2B: protein tyrosine kinase 2 beta
NFKB1: nuclear factor of kappa light polypeptide gene enhancer in B-cells 1, NF-kappaB
NFKBIA: nuclear factor of kappa light polypeptide gene enhancer in B-cells inhibitor, alpha, IkappaBalpha, IκB
H2AFX: H2A histone family, member X, H2AX
PML: promyelomonocytic leukemia
PML-NBs: promyelomonocytic leukemia nuclear bodies
ATM: ataxia telangiectasia mutated kinase
TP53: tumor protein p53
TOPBP1: topoisomerase IIβ-binding protein 1
NBN: Nijmegen breakage syndrome 1, NBS1
TP53BP1: tumor suppressor p53-binding protein 1, 53BP1
CHEK1 and CHEK2: checkpoint kinases 1 and 2
mTOR: mammalian target of rapamycin
MAPK14: mitogen-activated protein kinase 14, also called p38-α
MAP2K3: MAP kinase kinase 3
MAPK6: MAP kinase kinase 6
AIFM1: mitochondrion-associated apoptosis inducing factor 1 or AIF
TGM2: transglutaminase 2
BAX: BCL2-associated X protein
BAK1: BCL2-antagonist/killer 1
BBC3: BCL2 binding component 3 or PUMA
CYCS: cytochrome c, somatic
APAF1: apoptotic peptidase activating factor 1
CASP3: caspase-3
