# Supplementary material for: Syncytial apoptosis signaling network induced by the HIV-1 envelope glycoprotein complex: an overview
Source: Cell Death Dis. 2015 Aug 6;6(8):e1846–. doi: 10.1038/cddis.2015.204 (PMC4558497; doi:10.1038/cddis.2015.204)
Supplement: Supplementary Information [file cddis2015204x1.doc]

**Syncytial Apoptosis Map**

We used the software CellDesigner (Matsuoka *et al*, 2014) to build an annotated regulatory map (*cf* Supplementary Information: “SyncytialApoptosisMap_23012015.xml”) representing the reactions involved in the HIV-1-dependent formation of syncytia and the consequent induction of apoptosis. The map currently encompasses 36 components (proteins/genes) and 54 interactions. It covers the sequential activation of three main steps (from top to down):

1. The top of the map represents the interaction between a HIV-1 infected cell (purple compartment) and a CD4^+^ healthy cell (green compartment). This part includes all the reactions leading to the activation of P2RY2 and PTK2B proteins, determining cell-to-cell fusion.
2. The central part of the map represents all the events occurring within a pre-karyogamic syncytium (orange compartment). In particular, activation of NFKB1 and MTOR pathways are reported as main determinants of nuclear fusion (karyogamy).
3. The bottom of the map represents the reactions causing apoptosis in a post-karyogamic syncytium (grey compartment with dark background). Activated TP53 protein is the main actor of this step, leading to Mitochondrial Membrane Permealisation and Apoptosome formation, and subsequently to Syncytial Apoptosis.

The main compartments of the map thus represent different cellular states. In each case, sub-cellular compartments are indicated when needed: nuclei are denoted with blue contours, whereas mitochondria (only in the post-karyogamic syncytium) with red contours. Proteins are coloured in green, genes are coloured in yellow, viruses are coloured in purple.

A navigable version of the Syncytial Apoptosis Map, obtained using NaviCell web-based environment (Kuperstein *et al*, 2013), can be accessed at:

<http://syncytialapoptosismap.altervista.org/maps/SyncytialApoptosisMap/master/index.html>.

**References**

Kuperstein I, Cohen DPA, Pooks S, Viara E, Calzone L, Barillot E and Zinovyev A (2013) NaviCell: a web-based environment for navigation, curation and maintenance of large molecular interaction maps. *BMC Syst Biol* 7(1):100

[Matsuoka Y](http://www.ncbi.nlm.nih.gov/pubmed/?term=Matsuoka Y%5BAuthor%5D&cauthor=true&cauthor_uid=24927840), [Funahashi A](http://www.ncbi.nlm.nih.gov/pubmed/?term=Funahashi A%5BAuthor%5D&cauthor=true&cauthor_uid=24927840), [Ghosh S](http://www.ncbi.nlm.nih.gov/pubmed/?term=Ghosh S%5BAuthor%5D&cauthor=true&cauthor_uid=24927840), [Kitano H](http://www.ncbi.nlm.nih.gov/pubmed/?term=Kitano H%5BAuthor%5D&cauthor=true&cauthor_uid=24927840) (2014) Modeling and simulation using CellDesigner. *Methods Mol Biol* 1164:121-45

**Map notation**

**
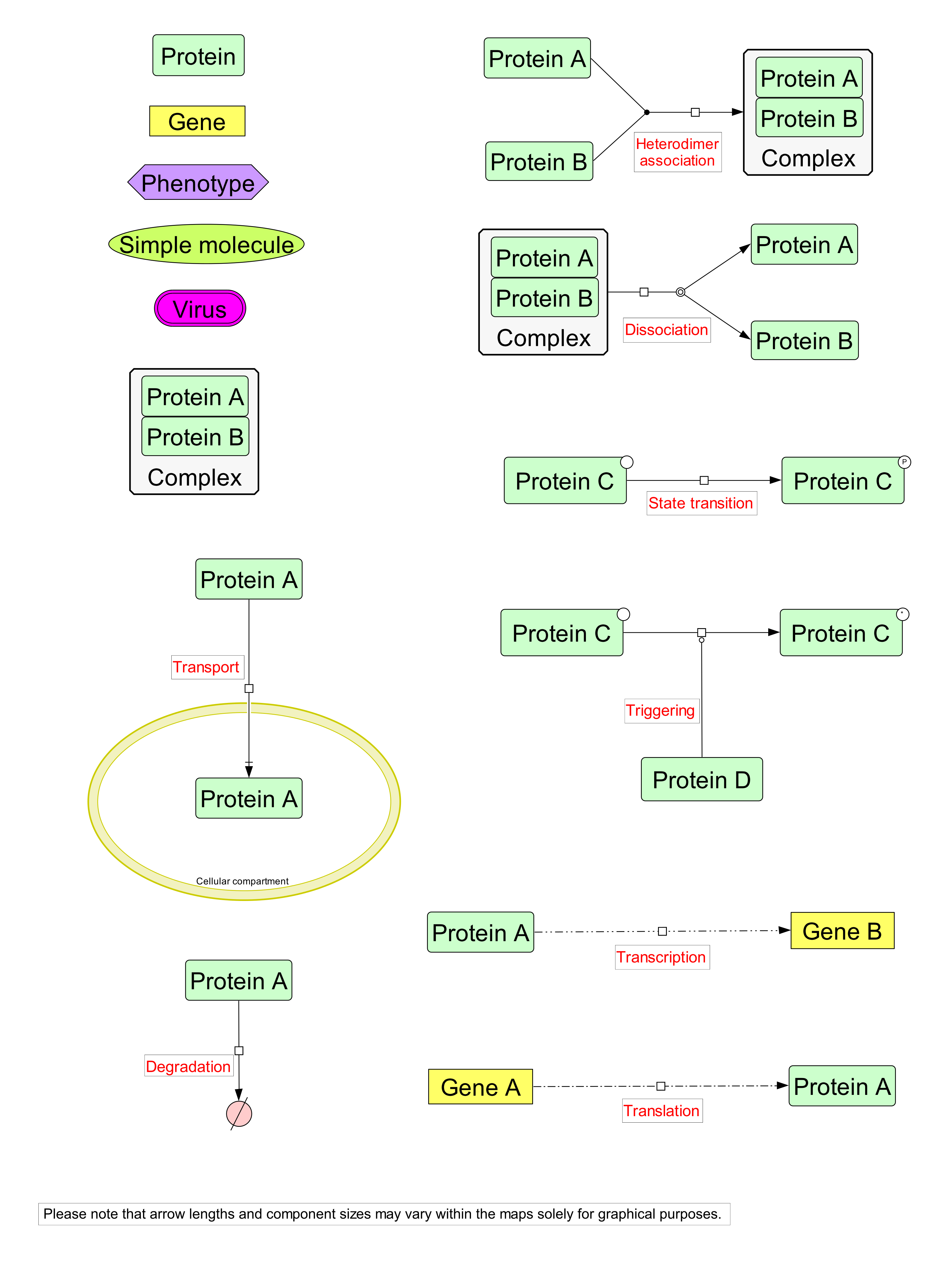
**

**Short tutorial on the use of the Syncytial Apoptosis Map (web version)**

Web link:

<http://syncytialapoptosismap.altervista.org/maps/SyncytialApoptosisMap/master/index.html>

NB: successfully tested with Mozilla Firefox web browser

**Browsing the map**

At the top left of the page you can choose the zoom level (there are 4 zoom levels). You can browse the map by clicking and dragging your mouse.

**Retrieval of protein information (example on how to visualise PUMA information)**

The column at the right hand side of the web page reports the list of all the map entities and reactions. Let us suppose you are interested in finding the information related to the protein PUMA:

- Click on the little triangle corresponding to “Proteins” section in the column: the list of all the proteins in the map will appear.
- Put a thick on the “BBC3” square: all BBC3 proteins present in the map will be pointed by marks (just as in Google Map).
- You will have BBC3 “alone” and BBC3 in complexes; to find BBC3-related information, click on the mark pointing at the “alone” protein: a section with HGNC link, notes and references (PubMed IDs) will appear.
- Click on the links to have more detailed information about BBC3 and its involvement in syncytial apoptosis.

**Retrieval of reaction information**

Reaction IDs are also indicated in the map (as reaction labels). They can be used to retrieve information about a reaction of interest:

- Click on the little triangle corresponding to “Reactions” section in the column at the right hand side of the web page.
- Put a thick on the ID (“re1”, “re2”, etc.) corresponding to the reaction of interest in the map.
- A mark will appear on the map pointing to the reaction. To visualise reaction information, click on the mark: annotations and references (PubMed IDs) will appear.
- Click on the links to have more detailed information about the reaction.
